# Platelets and the Role of P2X Receptors in Nociception, Pain, Neuronal Toxicity and Thromboinflammation

**DOI:** 10.3390/ijms23126585

**Published:** 2022-06-13

**Authors:** Elias Rawish, Harald F. Langer

**Affiliations:** 1 Cardioimmunology Group, Medical Clinic II, University Heart Center Lübeck, 23538 Lübeck, Germany; elias.rawish@uksh.de; 2 University Hospital Schleswig-Holstein, Department of Cardiology, University Heart Center Lübeck, 23538 Lübeck, Germany; 3 DZHK (German Centre for Cardiovascular Research), Partner Site Hamburg/Lübeck/Kiel, 23562 Lübeck, Germany

**Keywords:** inflammation, P2X receptor, thrombosis, platelets, stroke, Alzheimer’s disease, Parkinson’s disease

## Abstract

P2X receptors belong to a family of cation channel proteins, which respond to extracellular adenosine 5′-triphosphate (ATP). These receptors have gained increasing attention in basic and translational research, as they are central to a variety of important pathophysiological processes such as the modulation of cardiovascular physiology, mediation of nociception, platelet and macrophage activation, or neuronal–glial integration. While P2X1 receptor activation is long known to drive platelet aggregation, P2X7 receptor antagonists have recently been reported to inhibit platelet activation. Considering the role of both P2X receptors and platelet-mediated inflammation in neuronal diseases such as multiple sclerosis, Alzheimer’s disease, Parkinson’s disease, and stroke, targeting purinergic receptors may provide a valuable novel therapeutic approach in these diseases. Therefore, the present review illuminates the role of platelets and purinergic signaling in these neurological conditions to evaluate potential translational implications.

## 1. The P2X Receptor Family

**Figure 1:**
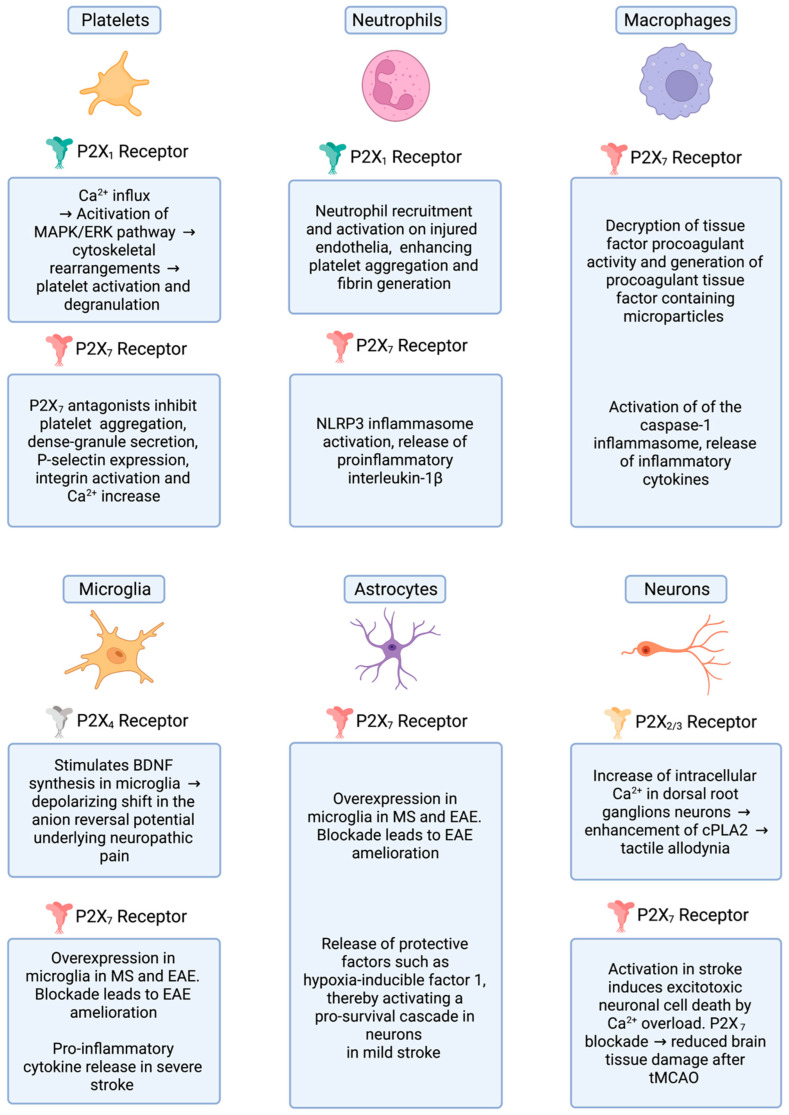
The role of P2X receptors in different cell types.

The ATP-gated P2X receptor cation channel family consists of a family of seven cation-permeable ligand-gated ion channels (P2X_1_R–P2X_7_R) that respond to extracellular adenosine 5’-triphosphate (ATP). The receptors are generally homo-trimeric or heterotrimeric, whereby each P2X subunit contains two transmembrane domains: an intracellular amino and carboxy termini and a large extracellular ligand-binding loop [1]. The attachment of ATP to an extracellular domain elicits a shift of the subunits, thereby separating the membrane-spanning region, which causes opening of the central channel [2]. P2X receptors have been identified in numerous human tissues and differ in their kinetics of desensitization by pharmaceuticals, which is largely determined by its subunit makeup [3]. However, all of them are activated by their physiological ligand ATP [4]. Notably, ATP has been described to play a decisive role in inflammation due to its release by damaged or injured cells [5]. Once in the extracellular milieu, ATP can bind to purinergic receptors, leading to the subsequent activation of several different signaling cascades. The functional involvement of P2X receptors in several physiological processes has been reported, including the regulation of vascular tone by being expressed in smooth muscle and endothelial cells [6], the contraction of the vas deferens during ejaculation [7], activation of macrophages [8] as well as the induction of macrophage apoptosis [9], platelet aggregation [10], neuromodulation [11], and nociceptive transmission [12]. Considering the variety of recent findings, it is impossible to cover all their functions in one review. Here, we focus on latest novelties concerning the role of platelets and P2X receptors in neuroinflammatory diseases to highlight novel implications for therapy and forge a translational bridge to clinical application, elucidating the capability of P2X receptors as treatment targets in these neuronal diseases such as multiple sclerosis, Alzheimer’s disease, Parkinson’s disease, stroke and neuropathic pain (Figure 1).

## 2. Regulation of Platelet Function by Purinergic Receptors

Thrombocytes—also termed platelets—are typically regarded as the key player of primary hemostasis. Ensuing endothelial injury, they prevent and halt bleeding by quick attachment to damaged vessels, thereby contributing to the formation of thrombi [13]. Under flow conditions, the early bond of platelets to the injured blood vessel wall obliges the interaction between immobilized von Willebrand factor (vWF) on the endothelial surface or in the subendothelial matrix with its platelet receptor Glycoprotein (GP) Ibα, which is a part of the GPIb-IX-V complex [14,15]. Furthermore, exposed subendothelial collagen attaches reversibly to the platelet GPIa/IIa receptor (also called integrin α_2_β_1_) and the GPVI receptor, which is a member of the immunoglobulin superfamily [16]. The stable attachment of collagen to the platelet GPVI receptor enables endurance toward high shear rates and stimulates platelet activation by an increase in cytosolic Ca^2+^ concentration. Hence, the platelet shape shifts, and vWF, P-selectin, fibrinogen, and platelet endothelial adhesion molecule-1 (PECAM-1) from α-granules as well as ADP, ATP, serotonin, and calcium from dense granules are discharged, which in turn promotes additional platelets to be activated by autocrine and paracrine signaling [17,18]. The final common pathway of platelet activation constitutes the conformational change in platelet GPIIb/IIIa receptor (integrin α_IIb_β_3_), which induces the cross-link of vWF or fibrinogen between GPIIb/IIIa receptors, causing platelet aggregation [19].

Within this cascade, the P2X_1_ receptor supports platelet shape change by inducing MAPK/ERK pathway-dependent myosin light chain kinase activation-mediated cytoskeletal rearrangements, thus contributing to shear-induced platelet aggregation and degranulation during vWF-triggered platelet activation [20,21]. Indeed, P2X_1_^−/−^ mice exhibited resistance to thromboembolism induced by collagen and adrenaline injection as well as to thrombosis caused by localized laser-induced injury of the vessel wall of mesenteric arteries [20]. Accordingly, application of the P2X_1_ antagonist NF449 confirmed the antithrombotic potential of P2X_1_ inhibition [22]. Oury et al. have generated transgenic mice overexpressing the P2X_1_ receptor in the megakaryocytic cell line [23]. Platelets from these mice exhibited a more prominent P2X_1_-mediated Ca^2+^ influx and platelet shape change. Furthermore, an increase in fatal pulmonary thromboembolism was observed in transgenic mice overexpressing P2X_1_ compared to wild-type mice [23]. Interestingly, P2X_1_^−/−^ mice displayed no prolongation of bleeding time as compared with wild-type animals [20], indicating that the P2X_1_ receptor could serve as a potential target for safe antiplatelet drugs [24]. Underscoring a potential clinical relevance, P2X_1_ receptors were shown to mediate the activation of aspirin-treated platelets by thrombin and epinephrine [25].

Notably, P2X_7_ receptor signaling has been reported to contribute to tissue factor (TF)-dependent thrombosis in mice, as the stimulation of P2X_7_ receptors on macrophages and vascular smooth muscle cells (VSMC) was found to induce the decryption of TF procoagulant activity and the generation of procoagulant TF-containing microparticles [26]. Additionally, P2X_7_ antagonists AZD9056 and entecavir have recently been reported to inhibit platelet activation by preserving mitochondrial function, improving lipid peroxidation and increasing antioxidant activity [27]. In accordance, entecavir inhibited platelet aggregation, dense-granule secretion, P-selectin expression, integrin activation and Ca^2+^ increase [27], underscoring the potential of P2X_7_ inhibition in order to modulate platelet function.

**Figure 2:**
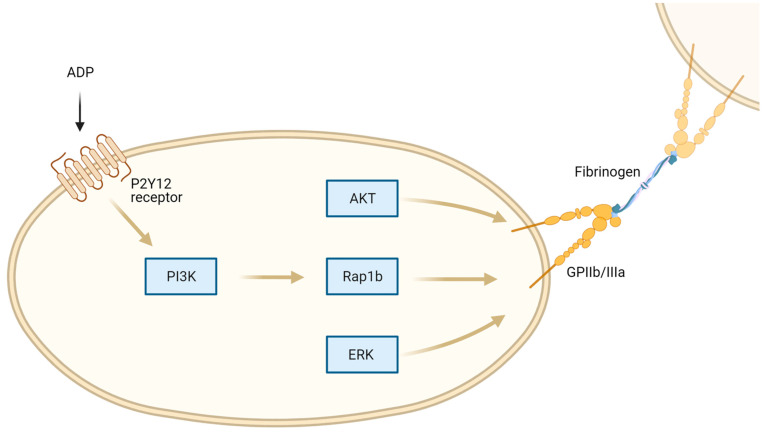
P2Y_12_ receptor in platelet activation. ADP binding to P2Y_12_ receptor leads to Phosphoinositide 3-kinase (PI3K) activation and subsequent glycoprotein (GP) IIb/IIIa activation via AKT, Ras-related protein (Rap-1b, and extracellular-signal regulated kinase (ERK) pathways.

With respect to purinergic signaling in platelet activation, P2Y_12_ receptors are crucial to mention, as P2Y_12_ antagonists such as clopidogrel, prasugrel, and ticagrelor are already widely used in cardiovascular diseases due to their potent antiplatelet effects. Briefly, ADP can bind Gi-coupled P2Y_12_ receptors and subsequently activate phosphoinositide 3-kinase (PI3K), Akt, extracellular signal-regulated kinase (ERK) and Rap1b, which finally leads to GPIIb/IIIa activation amplifying further platelet stimulation (Figure 2) [28].

## 3. Platelet-Mediated Inflammation in MS/EAE

Beyond their importance in hemostasis and thrombosis, an increasing body of evidence points to a decisive role of platelets for (neuro)inflammatory and immune responses [29,30,31]. Neuroinflammation has been related to a variety of diseases including amyotrophic lateral sclerosis (ALS), traumatic brain injury, epilepsy, and Huntington’s chorea [32] but also with non-neurological chronic conditions such as obesity and diabetes [33]. While the contribution of platelets to central nervous system (CNS) inflammation in some of these diseases has recently been reviewed elsewhere [34], this part of the review focuses on multiple sclerosis (MS) and experimental autoimmune encephalomyelitis (EAE), which is a counterpart of MS in mice. MS is a neurodegenerative disease accompanied by chronic demyelination. Although the pathogenesis of MS is still not entirely understood, it is generally recognized as a diverse, immune-mediated disorder caused by environment–gene interactions [35]. Regions of demyelination (so called plaques) represent a decisive pathological hallmark of MS. These areas do characteristically display a disruption of the blood–brain barrier (BBB). This breach allows antigen-presenting cells (APCs) such as myeloid cells (dendritic cells, macrophages, microglia) and B cells to passage through the BBB and subsequently trigger the differentiation of memory T cells into pro-inflammatory T helper lymphocytes (Th1 and Th17). Endothelial and leukocyte adhesion molecules mediate the following recruitment of inflammatory effector cells into the CNS parenchyma, which is accompanied by the stimulation of microglia cells, which are the cellular mediators of the destruction of axonal myelin sheath [35].

Interestingly, platelet-specific GPIIb (CD41) has been detected in the plaques of MS patients as well as in the brain tissue of mice with EAE [36,37]. Correspondingly, cerebrospinal fluid levels of platelet-activating factor have been associated with both MS [38] and EAE [39] disease activity. Remarkably, platelet-activating factor receptor knockout yields a decrease in inflammation and demyelination in a mice EAE model [39]. However, the decisive contribution of platelets to EAE pathogenesis has been demonstrated as platelet depletion alleviated EAE in mice, mainly in the effector phase of the disease, thus diminishing CNS mRNA levels of IL-1β, chemokine (C-C motif) ligand 2 (CCL-2), CCL-5, CCL-19, and CD184 as well as the expression of intercellular adhesion molecule (ICAM)-1 [37]. Accordingly, the recruitment of leukocytes to the inflamed CNS was reduced by platelet depletion [37,40]. Moreover, EAE was ameliorated by antibodies against GPIIb/IIIa and platelet GPIb [37]. Interestingly, the P2Y_12_ receptor antagonists ticagrelor and clopidogrel were lately shown to diminish the disease severity of EAE in mice [41]. P2X_1_ antagonists, however, have not been investigated in the context of MS/EAE yet. Instead, a P2X_7_ receptor antagonist decreased astrogliosis as well as demyelination, and it improved neurological symptoms in an EAE rodent model [42]. Indeed, augmented levels of P2X_7_ receptor expression have been detected in microglia, astrocytes, and oligodendrocytes of multiple sclerosis patients [43,44,45]. Considering the aforementioned effect of both P2X_1_ and P2X_7_ antagonists on platelet aggregation, dense-granule secretion, and integrin activation, the role of P2X receptors in platelet activity in the context of EAE should be elucidated by further studies, as they may embody promising targets for future MS therapy.

Furthermore, Zabala et al. have recently demonstrated that blockade of the P2X_4_ receptor signaling worsens clinical signs in an EAE model by microglia activation and inhibition of myelin phagocytosis [46]. On the contrary, the potentiation of P2X_4_ receptor signaling by the allosteric modulator ivermectin caused a switch in microglia to an anti-inflammatory phenotype, enhancing myelin phagocytosis and ameliorating clinical signs of EAE [46]. Thus, therapeutic prospects of P2X_4_ receptor allosteric modulators in MS and other neuroinflammatory conditions remain to be explored in translational approaches.

### P2X Receptors in Familial Multiple Sclerosis

Evidence reveals an interaction of several genetic and environmental factors contributing to the development of MS. The influence of genetic factors is apparent in monozygotic and dizygotic twins. Furthermore, according to various family studies, the incidence of MS is higher in siblings and in close relatives of the patients, as 15% to 20% of the patients with MS do have a relative suffering the disease. Interestingly, functional variants in genes for P2X_7_ receptor and P2X_4_ receptor have recently been shown to modulate MS susceptibility. Applying sequencing analysis of P2X_4_ receptor and P2X_7_ receptor in 193 MS patients and 100 controls, Sadovnick et al. were able to identify a three-variant haplotype (P2X_7_ receptor rs140915863:C > T (p.T205M), P2X_7_ receptor rs201921967:A > G (p.N361S) and P2X_4_ receptor rs765866317:G > A (p.G135S)) separating with disease in a multi-incident family with MS. The authors demonstrated a reduction in phagocytic ability by functional analysis of this haplotype in HEK293 cells. Furthermore, Oyanguren-Desez et al. have discovered that the T allele of rs17525809 polymorphism, which yields an Ala-76 to Val change in the extracellular domain of P2X_7_ receptor, is more frequent in MS patients than in controls. Fascinatingly, P2X_7_ receptor variants with Val display a gain-of-function by showing higher calcium permeability, larger electrophysiological responses, and higher ethidium uptake. This effect is accompanied by an enhancement of the gain-of-function His-155 to Tyr substitution (rs208294) in the haplotype formed by these two variants. Thus, illuminating the role of P2X receptor polymorphisms may help to identify the genetic background predisposing for multiple sclerosis and its pathophysiology.

## 4. Platelet Activation in Alzheimer’s Disease

Alzheimer’s disease (AD) is a the most common neurodegenerative illness. The global prevalence is estimated to be as high as 50 million and is predicted to reach 152 million by 2050 [47]. AD causes cognitive impairment as the disease progresses. The neuropathological hallmarks of AD are the formation of intracellular neurofibrillary tangles and the deposition of amyloid-ß (Aß) in brain tissue and cerebral vessels (so-called cerebral amyloid angiopathy, CAA), accompanied by neuroinflammation as well as neuronal and synaptic loss. Notably, platelets secrete both Aß peptide and amyloid precursor protein (APP) following platelet activation [48,49], constituting the main source for Aß peptide and APP in the bloodstream [50,51]. There is evidence to suggest that both Aß and APP play a role in regulating thrombosis and hemostasis [52,53].

AD patients were found to have enhanced platelet activation already two decades ago [54]. This was later linked to an increase in lipid peroxidation [55]. Platelets have since been observed to have enhanced activity and increased adhesion to subendothelial matrix components in a transgenic mice model of AD [56,57]. Underscoring a pathophysiological significance of platelets in AD, ß-secretase, which is required for the cleavage of APP, has been exposed to be raised in circulating platelets of AD patients compared to controls [58].

Aggregated platelets were revealed as a first pathological sign in AD mouse model prior to Aß plaque formation, proposing platelets as a therapeutic target in early AD [59]. Synthetic monomeric Aβ_40_ binds through its Arg-His-Asp-Ser sequence to GPIIb/IIIa, stimulating ADP and chaperone protein clusterin secretion from platelets [60]. Furthermore, the formation of fibrillar Aβ aggregates and further Aβ_40_ binding to platelets, constituting a feed-forward loop, were observed [60].

Pointing to antiplatelet drugs as potential therapeutic targets in CAA and AD treatment, it has been revealed that the P2Y_12_ receptor antagonist clopidogrel inhibits Aβ aggregation in platelet cultures, which was accompanied by reduction in clusterin in the circulation and a diminished incidence of CAA in a transgenic mice AD model [60]. Indeed, platelets isolated from AD mice promote severe vessel damage, matrix metalloproteinases activation and neuroinflammation in wild-type mice brain in an organotypic ex vivo brain slice model, thereby inducing Aß-like immunoreactivity at the damaged vessel sites [61].

With respect to the role of P2X receptors in Alzheimer’s disease, a significant increase in the mRNA of P2X_1, 2, 5, 7_ receptors was found following 12 h of exposure of hippocampal neurons to Aβ; after 24 h, only P2X_2_ remained enhanced [62]. However, P2X_7_ is the P2X receptor most studied on the pathogenesis of AD [63]. The first observation of P2X_7_ receptors’ potential involvement in AD based on the upregulation of P2X_7_ receptor in microglial cells close to senile plaques both in AD patients and animal AD models [64,65,66]. Indeed, the P2X_7_ receptor was shown to be involved in amyloidogenic APP processing [67], synaptic failure and neuronal dyshomeostasis [62] as well as neuroinflammation [68] associated to AD. Accordingly, both P2X_7_ receptor blockade or depletion yield an improvement of neuropathological hallmarks and symptoms in an AD rodent model [66,69,70,71]. Thus, the P2X_7_ receptor should be further elucidated in translational approaches for the treatment of Alzheimer’s disease also in regard to platelets’ role in AD and the implication of P2X_7_ receptor signaling for platelet function.

## 5. Purinergic Receptors in Parkinson’s Disease

Parkinson’s disease (PD) is the second most common neurodegenerative disease. Its incidence is about 4% of the population over 80 years old [72]. The clinical symptoms of this motor disease include tremor of extremities, muscular rigidity, postural imbalance, and bradykinesia [73]. The neuropathology of PD is characterized by neuronal loss in the substantia nigra pars compacta (SNc), which leads to striatal dopamine deficiency, and intracellular inclusions of α-synuclein aggregates. The underlying molecular pathogenesis involves multiple pathways and mechanisms such as α-synuclein proteostasis, calcium homeostasis, mitochondrial dysfunction, axonal transport, oxidative stress, and neuroinflammation [73]. To date, L-Dopa or dopamine agonists constitute the most common pharmacological agents used in therapy. However, the long-term use of L-dopa or dopamine agonists leads to a loss of efficacy as dose augmentation is necessary, triggering side effects such as dyskinesia or psychological symptoms [74], underscoring the importance of development novel pharmacological approaches in PD treatment.

Interestingly, P2X_7_ receptor blockade by A-438059 was shown to prevent or reverse hemiparkinsonian symptoms in animals treated with the neurotoxin 6-hydroxydopamine (6-OHDA), which mimics PD pathology [75]. In addition, also P2X_7_ receptor antagonist Brilliant Blue G (BBG) was shown to prevent hemiparkinsonian behavior after 6-OHDA lesion by a combined control of synaptotoxicity, neurotoxicity and gliosis [76]. A more recent study showed a reversal effect of BBG treatment in 6-OHDA lesioned rodents as well [77].

Enhanced ATP release due to neuronal breakdown and consequent purinergic receptors activation was shown to cause intracellular α-synuclein accumulation in neighboring healthy neurons mediated by lysosome dysfunction [78]. Interestingly, P2X_1_ receptor antagonism or genetic depletion reduced α-synuclein aggregation induced by ATP released by dying neuronal cells in vitro [78]. Of note, P2X_7_ receptor blockade was not able to reduce α-synuclein aggregation following ATP release [78].

However, P2X_1_ receptor activation may contribute to α-synuclein aggregation, which in turn modulates P2X_7_ receptor activity, reactive oxygen species (ROS) production and, conclusively, ATP release [79]: ATP release triggered by α-synuclein was shown to activate P2X_7_ receptor [80]. Moreover, Jiang et al. showed that microglial cells challenged with α-synuclein presented increased ROS production through P2X_7_ receptor activation [81]. Therefore, both targeting P2X_1_ and P2X_7_ receptor may be promising pharmacological targets for translational approaches in PD treatment.

With respect to platelets, alterations in the ultrastructure, mitochondrial dysfunction, and increased glutamate uptake have been observed in patients with PD [82,83,84]. Furthermore, an increase in mean platelet volume of PD patients has been observed [85]. However, the role of platelets purinergic signaling in PD remains elusive yet.

## 6. P2X Receptors, Thromboinflammation and Brain Ischemia

Stroke constitutes the second leading cause of death worldwide [86]. The majority (80%) of all strokes are caused by cerebral ischemia [86]. In particular, non-lacunar ischemic strokes are generally of thromboembolic origin. Common sources of embolism are extracranial large artery atherosclerosis and atrial fibrillation [87]. The backbone of treatment for ischemic stroke is rapid recanalization by thrombolysis or thrombectomy [88]. However, many patients suffer secondary infarct growth despite successful vessel recanalization. This so-called reperfusion injury is attributed to the thromboinflammatory activity of platelets and immune system cells [31,89]. Particularly, T cells have been implicated to contribute to cerebral reperfusion injury, as immunodeficient Rag1^−/−^ mice, which are lacking T cells and B cells, developed smaller cerebral infarcts after transient middle cerebral artery occlusion (tMCAO) compared with WT mice [90,91]. Furthermore, the adoptive transfer of T cells to Rag1^−/−^ mice reconstituted vulnerability to reperfusion injury [90,91]. Indeed, forkhead box P3 (FOXP3)-positive regulatory T (T_reg_) cells were recognized as the harmful type of T cells in ischemia–reperfusion injury [92]. Interestingly, platelets depletion in Rag1^−/−^ mice that received an adoptive transfer of T_reg_ cells reduces infarct size at the level as in naive Rag1^−/−^ mice following tMCAO [92], thus underlining the decisive role of platelets in stroke-associated thromboinflammation. Indeed, targeting initial steps of platelet adhesion and activation (e.g., GPVI-collagen, GPIb-vWF) were shown to reduce infarct size in mice [31].

P2X_1_ receptors are not only expressed by platelets but also by neutrophils, thereby promoting neutrophil chemotaxis [93]. Neutrophils were shown to play a pivotal role in reperfusion injury during ischemic stroke as well [94,95]. In a laser-induced injury mouse model of thrombosis, ATP-activated neutrophils accumulated at the site of injury before platelets, contributing to the initiation of thrombosis [96]. Both pharmacological antagonization and P2X_1_ deficiency reduced neutrophil recruitment and activation on inflamed arteriolar endothelia, which was accompanied by an impairment of platelet aggregation and fibrin generation [96]. Fibrin generation was restored by the application of wild-type neutrophils in P2X_1_^−/−^ mice, while the infusion of both wild-type platelets and neutrophils was mandatory for regular thrombus growth, underscoring the importance of P2X_1_ receptors on both platelets and neutrophils for thromboinflammation [4,96]. Therefore, targeting P2X_1_ receptors could constitute a novel therapeutic strategy to prevent local thromboinflammation during ischemic stroke by inhibit platelets but also amend neutrophil function to potentially alleviate reperfusion injury. However, more basic science studies are necessary to evaluate this potential therapeutical approach, as there are a lack of data about P2X_1_ blockade in ischemic stroke models.

P2X_7_ receptors’ relevance in cerebrovascular diseases should be emphasized as well. Energy deprivation following stroke leads to anoxic depolarization and the release of excitatory neurotransmitters, such as ATP [97,98,99]. Subsequent P2X_7_ activation induces excitotoxic glial and neuronal cell death by Ca^2+^ overload [100]. Indeed, the pharmacological antagonism of P2X_7_ yields reduced brain tissue damage after tMCAO [100], which was accompanied by a significant reduction in neuronal death, DNA cleavage and glial activation [101]. In addition to neuronal and glial excitotoxicity, the stimulation of neutrophil P2X_7_ receptors excites NLRP3 inflammasome activation, mediating the release of proinflammatory cytokines, such as IL-1β and IL-18, thereby enhancing ischemic damage [102,103].

However, in mild sublethal ischemic stroke, P2X_7_ receptors were shown to activate astrocytes without producing any detectable brain damage by promoting the release of protective factors, such as hypoxia-inducible factor 1 (HIF 1) and activating a pro-survival cascade in neurons [104]. In contrast, in severe ischemic stroke, P2X_7_ receptors lead to the aforementioned excitotoxic neuronal cell death, subsequent demyelination, astrocytic and microglial activation with proinflammatory cytokine release [105]. In the light of this dual role, further illumination of P2X_7_ receptor’s role in ischemic stroke—also by usage of allosteric modulators—is necessitated to elucidate its therapeutic value in neurovascular thromboinflammatory diseases.

## 7. P2X Receptors, Neuropathic Pain and Nociception

Chronic pain represents a vastly prevalent encumbering condition. Particularly, neuropathic pain is of distinct interest due to limited therapeutic options. Neupathic pain is commonly caused by damage or disease affecting the somatosensory nervous system. Central neuropathic pain is associated with spinal cord injury, multiple sclerosis, and some strokes [106]. In contrast, peripheral neuropathies are frequently triggered by metabolic disorders such as diabetes, HIV-related neuropathy, herpes zoster infection, immune disorders, toxins, or physical trauma [106]. Neuropathic pain is frequently accompanied by dysesthesia and tactile allodynia, which is a hypersensitivity to innocuous stimuli.

P2X_2_ and P2X_3_ receptors in primary sensory neurons and P2X_4_ receptors in the spinal dorsal horns are considered to be crucial players in pathological neuropathic pain generation and maintenance [107,108]. Electrophysiological analyses revealed that homomeric P2X_3_ as well as heteromeric P2X_2_ and P2X_3_ receptors (P2X_2/3_) constitute the major P2X receptors in primary sensory neurons [109,110,111]: the activation of P2X receptors by ATP causes an increase in intracellular Ca^2+^ in dorsal root ganglions neurons, thereby leading to the development of neuropathic pain [112]. In particular, extracellular ATP has been displayed to increase the level of phospho-cytosolic phospholipase A_2_ (cPLA_2_) in cultured dorsal root ganglions neurons [113]. Importantly, phospho-cPLA_2_ expression rises in dorsal root ganglion neurons following peripheral nerve injury, which has been correlated with the intensity of tactile allodynia [113]. A-317491, a potent and selective P2X_3_ and P2X_2/3_ receptor antagonist, significantly decreased the number of dorsal root ganglion neurons presenting the redistribution of phospho-cPLA_2_ and inhibited tactile allodynia after peripheral nerve injury in vivo [113].

Interestingly, platelet-activating factor (PAF), a part of the downstream signal cascade following cPLA_2_ activation, has been associated with the development of tactile allodynia, as a pharmacological blockade of PAF receptors was shown to reduce tactile allodynia after peripheral nerve injury [114]. Indeed, PAF receptor deficiency in mice yields a reduction in tactile allodynia after peripheral nerve injury and a suppression of tumor necrosis factor α (TNFα) IL-1β levels—which are well-known inflammatory cytokines associated with nociceptive hypersensitivity—in the injured dorsal root ganglions [114,115]. Even though platelet P2Y_12_ receptors have recently been shown to regulate Complete Freund’s Adjuvant (CFA)-induced chronic hyperalgesia as well as the associated local inflammatory response [116], the role of platelets P2X receptors in chronic pain remains elusive and thus should be considered in future studies.

Considering the P2X_4_ receptor, its upregulated expression has been reported after peripheral nerve injury in the ipsilateral spinal cord, particularly in hyperactive microglia but interestingly not in astrocytes or neurons [117]. The intraspinal administration of P2X_4_ receptor antisense oligodeoxynucleotide diminished the induction of P2X_4_ receptor and inhibited tactile allodynia after nerve injury [117]. Contrariwise, the intraspinal application of microglia in which P2X_4_ receptors had been induced and stimulated caused tactile allodynia in naive rats [117], underscoring the importance of P2X_4_ receptor activation in microglia for tactile allodynia. Indeed, microglial P2X_4_ receptor activation was demonstrated to fuel the synthesis of brain-derived neurotrophic factor (BDNF) from microglia [118], which binds to transmembrane tyrosine kinase B (TrkB) in secondary sensory neurons, causing a depolarizing shift in the anion reversal potential underlying neuropathic pain [119].

With respect to the cell type responsible for releasing ATP within the spinal cord after peripheral nerve injury, Masuda et al. were able to show that vesicular nucleotide transporter (VNUT) is necessary for exocytotic ATP release from spinal dorsal horn neurons, as the increase in spinal ATP and tactile allodynia is inhibited only in mice with the specific deletion of VNUT in dorsal horn neurons but not in mice with the specific deletion of VNUT in primary sensory neurons, microglia, or astrocytes after peripheral nerve injury [120].

## 8. Potential Translational and Clinical Applications

Based on the basic studies portrayed above, P2X receptors should receive attention as potential therapeutic targets of several diseases including thromboinflammatory and neuroinflammatory conditions as well as chronic neuropathic pain. Considering the first ones, clinical data are lacking. While P2Y_12_ receptor antagonists are well established in the clinical management of (cardio)vascular diseases such as acute myocardial infarction [121] or stroke [122], P2X receptors have not been clinically investigated in these conditions yet. However, regarding the delineated importance of platelets in neurovascular thromboinflammation as well as the antithrombotic potential of P2X_1_ inhibition, targeting platelet P2X_1_ receptors provides a valuable therapeutic approach for the future to develop novel antiplatelet drugs. Considering the aforementioned ability of neutrophiles to express P2X_1_ receptors, thereby promoting neutrophil chemotaxis during reperfusion injury during ischemic stroke, P2X_1_ receptor antagonism could be protective in stroke not only by inhibiting platelet mediated reperfusion injury but also cerebral inflammation caused by neutrophiles. With respect to the proinflammatory potential of P2X_7_ receptor by the release of inflammatory cytokines from peripheral macrophages, the therapeutic potential of P2X_7_ receptor blockade has been investigated in clinical trials addressing rheumatoid arthritis [123,124] and Crohn’s disease [125]. While there was no protective effect in case of rheumatoid arthritis, an improvement of symptoms in patients with moderate-to-severe Crohn’s disease has been reported. Further considering the ability of P2X_7_ receptor to activate neuroglia, centrally permeable P2X_7_ receptor antagonists such as JNJ-47965567 [126], Compound 7 [127], and A-438079 [128] should be evaluated for the experimental treatment of neuroinflammatory diseases such as MS.

Regarding acute and long-term sequels of COVID-19, elevated levels of extracellular ATP induced by SARS-CoV-2 infection may trigger the hyperactivation of P2X_7_ receptors enhancing consequent neuroinflammatory processes, as P2X_7_ receptor antagonism has been proposed as a promising strategy to treat psychiatric symptoms and neurodegenerative diseases of COVID-19 patients.

With respect to chronic pain, the P2X_4_ receptor blocker may serve as a novel therapeutic agent in neuropathic pain not only after the mentioned nerve injury but also in herpetic pain, as the selective P2X_4_ receptor antagonist, NP-1815-PX, was able to diminish tactile allodynia in a rodent model of herpes simplex virus type 1 caused pain [129]. Recently, the selective P2X_3_ and P2X_2/3_ receptor antagonist MK-7264 (gefapixant) has been reported to inhibit pain in rodent neuropathic sensitization models [130]. Furthermore, gefapixant was shown to reduce cough frequency by diminishing the reflex sensitivity of patients with refractory chronic cough (RCC) two phase III studies [131]. However, dose-dependent taste-related adverse events were reported [132]. Interestingly, the more selective P2X_3_ receptor antagonist eliapixant reduced cough frequency and severity as well, while taste-related side effects were lower at therapeutic doses than with the less selective P2X_3_ receptor antagonist gefapixant [133].

## 9. Conclusions

In conclusion, growing evidence suggests a crucial involvement of P2X receptors in both platelet and neutrophil-mediated thromboinflammation, microglia-mediated neuroinflammation as well as in neuropathic pain. While P2X_1_ receptor antagonists may be useful in thromboinflammatory diseases such as stroke, as they are known to drive platelet aggregation; more recently, P2X_7_ receptor antagonists were demonstrated to modulate platelet function as well. Regarding the illustrated role of P2X_7_ receptors and platelet-mediated inflammation in EAE/MS, Alzheimer’s disease, and Parkinson’s disease, targeting purinergic receptors may provide novel therapeutic approaches in these devastating diseases. Furthermore, the use of P2X_7_ receptor antagonists may be an attractive future therapeutic option for neurological sequelae of COVID-19.

Of note, P2X_3_ and P2X_4_ receptor antagonists may be useful in chronic pain, while P2X_3_ antagonists have already been shown to be effective in the treatment of patients suffering refractory chronic cough. Nevertheless, there are still a lack of clinical data considering P2X receptors’ therapeutic potential in many of the aforementioned diseases. Expanding our knowledge about P2X receptors could therefore help to reveal feasible therapeutic strategies against burdening chronic neurological conditions.

## Data Availability

Not applicable.

## References

[1] North R.A. (2016). P2X receptors. Philos. Trans. R. Soc. B Biol. Sci..

[2] Illes P., Müller C.E., Jacobson K.A., Grutter T., Nicke A., Fountain S.J., Kennedy C., Schmalzing G., Jarvis M.F., Stojilkovic S.S. (2021). Update of P2X receptor properties and their pharmacology: IUPHAR Review 30. Br. J. Pharmacol..

[3] Gever J.R., Cockayne D.A., Dillon M.P., Burnstock G., Ford A.P. (2006). Pharmacology of P2X channels. Pharmacol. P2X Channels.

[4] Oury C., Lecut C., Hego A., Wéra O., Delierneux C. (2014). Purinergic control of inflammation and thrombosis: Role of P2X1 receptors. Comput. Struct. Biotechnol. J..

[5] Dosch M., Gerber J., Jebbawi F., Beldi G. (2018). Mechanisms of ATP Release by Inflammatory Cells. Int. J. Mol. Sci..

[6] Wang L., Karlsson L., Moses S., Hultgårdh-Nilsson A., Andersson M., Borna C., Gudbjartsson T., Jern S., Erlinge D. (2002). P2 Receptor Expression Profiles in Human Vascular Smooth Muscle and Endothelial Cells. J. Cardiovasc. Pharmacol..

[7] Mulryan K., Gitterman D.P., Lewis C.J., Vial C., Leckie B.J., Cobb A.L., Brown J.E., Conley E.C., Buell G., Pritchard C.A. (2000). Reduced vas deferens contraction and male infertility in mice lacking P2X1 receptors. Nature.

[8] Wewers M.D., Sarkar A. (2009). P2X7 receptor and macrophage function. Purinergic Signal..

[9] Kawano A., Tsukimoto M., Noguchi T., Hotta N., Harada H., Takenouchi T., Kitani H., Kojima S. (2012). Involvement of P2X4 receptor in P2X7 receptor-dependent cell death of mouse macrophages. Biochem. Biophys. Res. Commun..

[10] Gachet C. (2008). P2 receptors, platelet function and pharmacological implications. Thromb. Haemost..

[11] Khakh B.S., North R.A. (2012). Neuromodulation by Extracellular ATP and P2X Receptors in the CNS. Neuron.

[12] Kuan Y.-H., Shyu B.-C. (2016). Nociceptive transmission and modulation via P2X receptors in central pain syndrome. Mol. Brain.

[13] Golebiewska E.M., Poole A.W. (2015). Platelet secretion: From haemostasis to wound healing and beyond. Blood Rev..

[14] Savage B., Saldívar E., Ruggeri Z.M. (1996). Initiation of Platelet Adhesion by Arrest onto Fibrinogen or Translocation on von Willebrand Factor. Cell.

[15] Schneider S.W., Nuschele S., Wixforth A., Gorzelanny C., Alexander-Katz A., Netz R.R., Schneider M.F. (2007). Shear-induced unfolding triggers adhesion of von Willebrand factor fibers. Proc. Natl. Acad. Sci. USA.

[16] Furie B., Furie B.C. (2005). Thrombus formation in vivo. J. Clin. Investig..

[17] Harrison P., Cramer E.M. (1993). Platelet alpha-granules. Blood Rev..

[18] Blair P., Flaumenhaft R. (2009). Platelet α-granules: Basic biology and clinical correlates. Blood Rev..

[19] Storey R., Thomas M.R. (2015). The role of platelets in inflammation. Thromb. Haemost..

[20] Hechler B., Lenain N., Marchese P., Vial C., Heim V., Freund M., Cazenave J.-P., Cattaneo M., Ruggeri Z.M., Evans R. (2003). A Role of the Fast ATP-gated P2X1 Cation Channel in Thrombosis of Small Arteries In Vivo. J. Exp. Med..

[21] Oury C., Sticker E., Cornelissen H., de Vos R., Vermylen J., Hoylaerts M.F. (2004). ATP augments von Willebrand factor-dependent shear-induced platelet aggregation through Ca2+-calmodulin and myosin light chain kinase activation. J. Biol. Chem..

[22] Hechler B., Cattaneo M., Gachet C. (2005). The P2 Receptors in Platelet Function. Semin. Thromb. Hemost..

[23] Oury C.C., Kuijpers M.J.E., Toth-Zsamboki E., Bonnefoy A., Danloy S., Vreys I., Feijge M.A.H., de Vos R., Vermylen J., Heemskerk J.W.M. (2003). Overexpression of the platelet P2X1 ion channel in transgenic mice generates a novel prothrombotic phenotype. Blood.

[24] Hechler B., Gachet C. (2015). Purinergic Receptors in Thrombosis and Inflammation. Arter. Thromb. Vasc. Biol..

[25] Grenegård M., Vretenbrant-Öberg K., Nylander M., Désilets S., Lindström E.G., Larsson A., Ramström I., Ramström S., Lindahl T.L. (2008). The ATP-gated P2X1 Receptor Plays a Pivotal Role in Activation of Aspirin-treated Platelets by Thrombin and Epinephrine. J. Biol. Chem..

[26] Furlan-Freguia C., Marchese P., Gruber A., Ruggeri Z.M., Ruf W. (2011). P2X7 receptor signaling contributes to tissue factor-dependent thrombosis in mice. J. Clin. Investig..

[27] Ming Y., Xin G., Ji B., Ji C., Wei Z., Zhang B., Zhang J., Yu K., Zhang X., Li S. (2020). Entecavir as a P2X7R antagonist ameliorates platelet activation and thrombus formation. J. Pharmacol. Sci..

[28] Kim S., Kunapuli S.P. (2011). P2Y12 receptor in platelet activation. Platelets.

[29] Mezger M., Nording H., Sauter R., Graf T., Heim C., Von Bubnoff N., Ensminger S.M., Langer H.F. (2019). Platelets and Immune Responses During Thromboinflammation. Front. Immunol..

[30] Rawish E., Sauter M., Sauter R., Nording H., Langer H.F. (2021). Complement, inflammation and thrombosis. Br. J. Pharmacol..

[31] Rawish E., Nording H., Münte T., Langer H.F. (2020). Platelets as Mediators of Neuroinflammation and Thrombosis. Front. Immunol..

[32] Gilhus N.E., Deuschl G. (2019). Neuroinflammation—A common thread in neurological disorders. Nat. Rev. Neurol..

[33] Rawish E., Nickel L., Schuster F., Stölting I., Frydrychowicz A., Saar K., Hübner N., Othman A., Kuerschner L., Raasch W. (2020). Telmisartan prevents development of obesity and normalizes hypothalamic lipid droplets. J. Endocrinol..

[34] Leiter O., Walker T.L. (2020). Platelets in Neurodegenerative Conditions-Friend or Foe?. Front. Immunol..

[35] Filippi M., Bar-Or A., Piehl F., Preziosa P., Solari A., Vukusic S., Rocca M.A. (2018). Multiple sclerosis. Nat. Rev. Dis. Primers.

[36] Lock C., Hermans G., Pedotti R., Brendolan A., Schadt E., Garren H., Langer-Gould A., Strober S., Cannella B., Allard J. (2002). Gene-microarray analysis of multiple sclerosis lesions yields new targets validated in autoimmune encephalomyelitis. Nat. Med..

[37] Langer H.F., Choi E.Y., Zhou H., Schleicher R., Chung K.J., Tang Z., Gobel K., Bdeir K., Chatzigeorgiou A., Wong C. (2012). Platelets contribute to the pathogenesis of experimental autoimmune encephalomyelitis. Circ. Res..

[38] Callea L., Arese M., Orlandini A., Bargnani C., Priori A., Bussolino F. (1999). Platelet activating factor is elevated in cerebral spinal fluid and plasma of patients with relapsing–remitting multiple sclerosis. J. Neuroimmunol..

[39] Kihara Y., Ishii S., Kita Y., Toda A., Shimada A., Shimizu T. (2005). Dual phase regulation of experimental allergic encephalomyelitis by platelet-activating factor. J. Exp. Med..

[40] D’Souza C.S., Li Z., Maxwell D.L., Trusler O., Murphy M., Crewther S., Peter K., Orian J.M. (2018). Platelets Drive Inflammation and Target Gray Matter and the Retina in Autoimmune-Mediated Encephalomyelitis. J. Neuropathol. Exp. Neurol..

[41] Qin C., Zhou J., Gao Y., Lai W., Yang C., Cai Y., Chen S., Du C. (2017). Critical Role of P2Y12 Receptor in Regulation of Th17 Differentiation and Experimental Autoimmune Encephalomyelitis Pathogenesis. J. Immunol..

[42] Grygorowicz T., Welniak-Kaminska M., Strużyńska L. (2016). Early P2X7R-related astrogliosis in autoimmune encephalomyelitis. Mol. Cell. Neurosci..

[43] Yiangou Y., Facer P., Durrenberger P., Chessell I.P., Naylor A., Bountra C., Banati R.R., Anand P. (2006). COX-2, CB2 and P2X7-immunoreactivities are increased in activated microglial cells/macrophages of multiple sclerosis and amyotrophic lateral sclerosis spinal cord. BMC Neurol..

[44] Narcisse L., Scemes E., Zhao Y., Lee S.C., Brosnan C.F. (2005). The cytokine IL-1beta transiently enhances P2X7 receptor expression and function in human astrocytes. Glia.

[45] Matute C., Torre I., Pérez-Cerdá F., Pérez-Samartín A., Alberdi E., Etxebarria E., Arranz A.M., Ravid R., Rodríguez-Antigüedad A., Sánchez-Gómez M. (2007). P2X(7) receptor blockade prevents ATP excitotoxicity in oligodendrocytes and ameliorates experimental autoimmune encephalomyelitis. J. Neurosci..

[46] Zabala A., Vazquez-Villoldo N., Rissiek B., Gejo J., Martin A., Palomino A., Perez-Samartín A., Pulagam K.R., Lukowiak M., Capetillo-Zarate E. (2018). P2X4 receptor controls microglia activation and favors remyelination in autoimmune encephalitis. EMBO Mol. Med..

[47] Breijyeh Z., Karaman R. (2020). Comprehensive Review on Alzheimer’s Disease: Causes and Treatment. Molecules.

[48] Li Q.X., Whyte S., Tanner J.E., Evin G., Beyreuther K., Masters C.L. (1998). Secretion of Alzheimer’s disease Abeta amyloid peptide by activated human platelets. Lab. Investig. J. Tech. Methods Pathol..

[49] Bush A.I., Martins R.N., Rumble B., Moir R., Fuller S., Milward E., Currie J., Ames D., Weidemann A., Fischer P. (1990). The amyloid precursor protein of Alzheimer’s disease is released by human platelets. J. Biol. Chem..

[50] Chen M., Inestrosa N.C., Ross G.S., Fernandez H.L. (1995). Platelets are the primary source of amyloid beta-peptide in human blood. Biochem. Biophys. Res. Commun..

[51] Li Q.X., Evin G., Small D.H., Multhaup G., Beyreuther K., Masters C.L. (1995). Proteolytic processing of Alzheimer’s disease beta A4 amyloid precursor protein in human platelets. J. Biol. Chem..

[52] Kowalska M.A., Badellino K. (1994). Beta-Amyloid protein induces platelet aggregation and supports platelet adhesion. Biochem. Biophys. Res. Commun..

[53] Visconte C., Canino J., Guidetti G.F., Zarà M., Seppi C., Abubaker A.A., Pula G., Torti M., Canobbio I. (2018). Amyloid precursor protein is required for in vitro platelet adhesion to amyloid peptides and potentiation of thrombus formation. Cell. Signal..

[54] Sevush S., Jy W., Horstman L.L., Mao W.-W., Kolodny L., Ahn Y.S. (1998). Platelet Activation in Alzheimer Disease. Arch. Neurol..

[55] Ciabattoni G., Porreca E., di Febbo C., di Iorio A., Paganelli R., Bucciarelli T., Pescara L., del Re L., Giusti C., Falco A. (2007). Determinants of platelet activation in Alzheimer’s disease. Neurobiol. Aging.

[56] Jarre A., Gowert N.S., Donner L., Munzer P., Klier M., Borst O., Schaller M., Lang F., Korth C., Elvers M. (2014). Pre-activated blood platelets and a pro-thrombotic phenotype in APP23 mice modeling Alzheimer’s disease. Cell. Signal..

[57] Canobbio I., Visconte C., Oliviero B., Guidetti G., Zara M., Pula G., Torti M. (2016). Increased platelet adhesion and thrombus formation in a mouse model of Alzheimer’s disease. Cell. Signal..

[58] Johnston J.A., Liu W.W., Coulson D.T., Todd S., Murphy S., Brennan S., Foy C.J., Craig D., Irvine G.B., Passmore A.P. (2008). Platelet beta-secretase activity is increased in Alzheimer’s disease. Neurobiol. Aging.

[59] Kniewallner K.M., Wenzel D., Humpel C. (2016). Thiazine Red+ platelet inclusions in Cerebral Blood Vessels are first signs in an Alzheimer’s Disease mouse model. Sci. Rep..

[60] Donner L., Falker K., Gremer L., Klinker S., Pagani G., Ljungberg L.U., Lothmann K., Rizzi F., Schaller M., Gohlke H. (2016). Platelets contribute to amyloid-beta aggregation in cerebral vessels through integrin alphaIIbbeta3-induced outside-in signaling and clusterin release. Sci. Signal..

[61] Kniewallner K.M., Foidl B.M., Humpel C. (2018). Platelets isolated from an Alzheimer mouse damage healthy cortical vessels and cause inflammation in an organotypic ex vivo brain slice model. Sci. Rep..

[62] Sáez-Orellana F., Fuentes-Fuentes M.C., Godoy P.A., Silva-Grecchi T., Panes J.D., Guzmán L., Yévenes G.E., Gavilán J., Egan T.M., Aguayo L.G. (2018). P2X receptor overexpression induced by soluble oligomers of amyloid beta peptide potentiates synaptic failure and neuronal dyshomeostasis in cellular models of Alzheimer’s disease. Neuropharmacology.

[63] Godoy P.A., Molina O.R., Fuentealba J. (2019). Exploring the Role of P2X Receptors in Alzheimer’s Disease. Front. Pharmacol..

[64] Parvathenani L.K., Tertyshnikova S., Greco C.R., Roberts S.B., Robertson B., Posmantur R. (2003). P2X7 mediates superoxide production in primary microglia and is up-regulated in a transgenic mouse model of Alzheimer’s disease. J. Biol. Chem..

[65] McLarnon J.G., Ryu J.K., Walker D.G., Choi H.B. (2006). Upregulated expression of purinergic P2X(7) receptor in Alzheimer disease and amyloid-beta peptide-treated microglia and in peptide-injected rat hippocampus. J. Neuropathol. Exp. Neurol..

[66] Ryu J.K., McLarnon J.G. (2008). Block of purinergic P2X(7) receptor is neuroprotective in an animal model of Alzheimer’s disease. Neuroreport.

[67] Delarasse C., Auger R., Gonnord P., Fontaine B., Kanellopoulos J.M. (2011). The purinergic receptor P2X7 triggers alpha-secretase-dependent processing of the amyloid precursor protein. J. Biol. Chem..

[68] Martínez-Frailes C., di Lauro C., Bianchi C., de Diego-García L., Sebastián-Serrano Á., Boscá L., Díaz-Hernández M. (2019). Amyloid Peptide Induced Neuroinflammation Increases the P2X7 Receptor Expression in Microglial Cells, Impacting on Its Functionality. Front. Cell. Neurosci..

[69] Chen X., Hu J., Jiang L., Xu S., Zheng B., Wang C., Zhang J., Wei X., Chang L., Wang Q. (2014). Brilliant Blue G improves cognition in an animal model of Alzheimer’s disease and inhibits amyloid-β-induced loss of filopodia and dendrite spines in hippocampal neurons. Neuroscience.

[70] Diaz-Hernandez J.I., Gomez-Villafuertes R., León-Otegui M., Hontecillas-Prieto L., del Puerto A., Trejo J.L., Lucas J.J., Garrido J.J., Gualix J., Miras-Portugal M.T. (2012). In vivo P2X7 inhibition reduces amyloid plaques in Alzheimer’s disease through GSK3β and secretases. Neurobiol. Aging.

[71] Martin E., Amar M., Dalle C., Youssef I., Boucher C., Le Duigou C., Brückner M., Prigent A., Sazdovitch V., Halle A. (2018). New role of P2X7 receptor in an Alzheimer’s disease mouse model. Mol. Psychiatry.

[72] De Lau L.M., Breteler M.M. (2006). Epidemiology of Parkinson’s disease. Lancet Neurol..

[73] Poewe W., Seppi K., Tanner C.M., Halliday G.M., Brundin P., Volkmann J., Schrag A.-E., Lang A.E. (2017). Parkinson disease. Nat. Rev. Dis. Primers.

[74] Salat D., Tolosa E. (2013). Levodopa in the treatment of Parkinson’s disease: Current status and new developments. J. Parkinsons Dis..

[75] Marcellino D., Suárez-Boomgaard D., Sánchez-Reina M.D., Aguirre J.A., Yoshitake T., Yoshitake S., Hagman B., Kehr J., Agnati L.F., Fuxe K. (2010). On the role of P2X7 receptors in dopamine nerve cell degeneration in a rat model of Parkinson’s disease: Studies with the P2X7 receptor antagonist A-438079. J. Neural Transm..

[76] Carmo M., Menezes A.P.F., Nunes A.C.L., Pliássova A., Rolo A., Palmeira C., Cunha R.A., Canas P., Andrade G. (2014). The P2X7 receptor antagonist Brilliant Blue G attenuates contralateral rotations in a rat model of Parkinsonism through a combined control of synaptotoxicity, neurotoxicity and gliosis. Neuropharmacology.

[77] Ferrazoli E.G., de Souza H.D.N., Nascimento I.C., Oliveira-Giacomelli Á., Schwindt T.T., Britto L.R., Ulrich H. (2017). Brilliant Blue G, but not Fenofibrate, Treatment Reverts Hemiparkinsonian Behavior and Restores Dopamine Levels in an Animal Model of Parkinson’s Disease. Cell Transplant..

[78] Gan M., Moussaud S., Jiang P., McLean P.J. (2015). Extracellular ATP induces intracellular alpha-synuclein accumulation via P2X1 receptor-mediated lysosomal dysfunction. Neurobiol. Aging.

[79] Oliveira-Giacomelli Á., Naaldijk Y., Sardá-Arroyo L., Gonçalves M.C.B., Corrêa-Velloso J., Pillat M.M., de Souza H.D.N., Ulrich H. (2018). Purinergic Receptors in Neurological Diseases With Motor Symptoms: Targets for Therapy. Front. Pharmacol..

[80] Wilkaniec A., Gąssowska M., Czapski G., Cieślik M., Sulkowski G., Adamczyk A. (2017). P2X7 receptor-pannexin 1 interaction mediates extracellular alpha-synuclein-induced ATP release in neuroblastoma SH-SY5Y cells. Purinergic Signal..

[81] Jiang T., Hoekstra J., Heng X., Kang W., Ding J., Liu J., Chen S., Zhang J. (2015). P2X7 receptor is critical in α-synuclein–mediated microglial NADPH oxidase activation. Neurobiol. Aging.

[82] Espinosa-Parrilla Y., Gonzalez-Billault C., Fuentes E., Palomo I., Alarcón M. (2019). Decoding the Role of Platelets and Related MicroRNAs in Aging and Neurodegenerative Disorders. Front. Aging Neurosci..

[83] Haas R.H., Nasirian F., Nakano K., Ward D., Pay M., Hill R., Shults C.W. (1995). Low platelet mitochondrial complex I and complex II/III activity in early untreated parkinson’s disease. Ann. Neurol..

[84] Bronstein J.M., Paul K., Yang L., Haas R.H., Shults C.W., Le T., Ritz B. (2015). Platelet mitochondrial activity and pesticide exposure in early Parkinson’s disease. Mov. Disord..

[85] Koçer A., Yaman A., Niftaliyev E., Dürüyen H., Eryılmaz M., Koçer E. (2013). Assessment of Platelet Indices in Patients with Neurodegenerative Diseases: Mean Platelet Volume Was Increased in Patients with Parkinson’s Disease. Curr. Gerontol. Geriatr. Res..

[86] Johnson C.O. (2019). Global, regional, and national burden of stroke, 1990–2016: A systematic analysis for the Global Burden of Disease Study 2016. Lancet Neurol..

[87] Campbell B.C.V., de Silva D.A., Macleod M.R., Coutts S.B., Schwamm L.H., Davis S.M., Donnan G.A. (2019). Ischaemic stroke. Nat. Rev. Dis. Primers.

[88] Powers W.J., Rabinstein A.A., Ackerson T., Adeoye O.M., Bambakidis N.C., Becker K., Biller J., Brown M., Demaerschalk B.M., Hoh B. (2019). Guidelines for the Early Management of Patients With Acute Ischemic Stroke: 2019 Update to the 2018 Guidelines for the Early Management of Acute Ischemic Stroke: A Guideline for Healthcare Professionals From the American Heart Association/American Stroke Association. Stroke.

[89] Nieswandt B., Kleinschnitz C., Stoll G. (2011). Ischaemic stroke: A thrombo-inflammatory disease?. J. Physiol..

[90] Yilmaz G., Arumugam T.V., Stokes K.Y., Granger D.N. (2006). Role of T lymphocytes and interferon-gamma in ischemic stroke. Circulation.

[91] Kleinschnitz C., Schwab N., Kraft P., Hagedorn I., Dreykluft A., Schwarz T., Austinat M., Nieswandt B., Wiendl H., Stoll G. (2010). Early detrimental T-cell effects in experimental cerebral ischemia are neither related to adaptive immunity nor thrombus formation. Blood.

[92] Kleinschnitz C., Kraft P., Dreykluft A., Hagedorn I., Goebel K., Schuhmann M.K., Langhauser F., Helluy X., Schwarz T., Bittner S. (2013). Regulatory T cells are strong promoters of acute ischemic stroke in mice by inducing dysfunction of the cerebral microvasculature. Blood.

[93] Lecut C., Frederix K., Johnson D.M., Deroanne C., Thiry M., Faccinetto C., Marée R., Evans R.J., Volders P.G.A., Bours V. (2009). P2X_1_ Ion Channels Promote Neutrophil Chemotaxis through Rho Kinase Activation. J. Immunol..

[94] Chou W.-H., Choi D.-S., Zhang H., Mu D., McMahon T., Kharazia V.N., Lowell C.A., Ferriero D.M., Messing R. (2004). Neutrophil protein kinase Cδ as a mediator of stroke-reperfusion injury. J. Clin. Investig..

[95] Chen R., Zhang X., Gu L., Zhu H., Zhong Y., Ye Y., Xiong X., Jian Z. (2021). New Insight Into Neutrophils: A Potential Therapeutic Target for Cerebral Ischemia. Front. Immunol..

[96] Darbousset R., Delierneux C., Mezouar S., Hego A., Lecut C., Guillaumat I., Riederer M.A., Evans R.J., Dignat-George F., Panicot-Dubois L. (2014). P2X1 expressed on polymorphonuclear neutrophils and platelets is required for thrombosis in mice. Blood.

[97] Melani A., Turchi D., Vannucchi M.G., Cipriani S., Gianfriddo M., Pedata F. (2005). ATP extracellular concentrations are increased in the rat striatum during in vivo ischemia. Neurochem. Int..

[98] Rossi D.J., Brady J.D., Mohr C. (2007). Astrocyte metabolism and signaling during brain ischemia. Nat. Neurosci..

[99] Braun N., Zhu Y., Krieglstein J., Culmsee C., Zimmermann H. (1998). Upregulation of the Enzyme Chain Hydrolyzing Extracellular ATP after Transient Forebrain Ischemia in the Rat. J. Neurosci..

[100] Arbeloa J., Pérez-Samartín A., Gottlieb M., Matute C. (2012). P2X7 receptor blockade prevents ATP excitotoxicity in neurons and reduces brain damage after ischemia. Neurobiol. Dis..

[101] Chu K., Yin B., Wang J., Peng G., Liang H., Xu Z., Du Y., Fang M., Xia Q., Luo B. (2012). Inhibition of P2X7 receptor ameliorates transient global cerebral ischemia/reperfusion injury via modulating inflammatory responses in the rat hippocampus. J. Neuroinflammation.

[102] Andrejew R., Oliveira-Giacomelli Á., Ribeiro D.E., Glaser T., Arnaud-Sampaio V.F., Lameu C., Ulrich H. (2020). The P2X7 Receptor: Central Hub of Brain Diseases. Front. Mol. Neurosci..

[103] Karmakar M., Katsnelson M.A., Dubyak G.R., Pearlman E. (2016). Neutrophil P2X7 receptors mediate NLRP3 inflammasome-dependent IL-1β secretion in response to ATP. Nat. Commun..

[104] Hirayama Y., Ikeda-Matsuo Y., Notomi S., Enaida H., Kinouchi H., Koizumi S. (2015). Astrocyte-Mediated Ischemic Tolerance. J. Neurosci..

[105] Cisneros-Mejorado A.J., Pérez-Samartín A., Domercq M., Arellano R.O., Gottlieb M., Koch-Nolte F., Matute C. (2020). P2X7 Receptors as a Therapeutic Target in Cerebrovascular Diseases. Front. Mol. Neurosci..

[106] Colloca L., Ludman T., Bouhassira D., Baron R., Dickenson A.H., Yarnitsky D., Freeman R., Truini A., Attal N., Finnerup N.B. (2017). Neuropathic pain. Nat. Rev. Dis. Primers.

[107] Bernier L.-P., Ase A.R., Séguéla P. (2018). P2X receptor channels in chronic pain pathways. J. Cereb. Blood Flow Metab..

[108] Inoue K. (2007). P2 receptors and chronic pain. Purinergic Signal.

[109] Rae M.G., Rowan E.G., Kennedy C. (1998). Pharmacological properties of P2X3-receptors present in neurones of the rat dorsal root ganglia. Br. J. Pharmacol..

[110] Ueno S., Tsuda M., Iwanaga T., Inoue K. (1999). Cell type-specific ATP-activated responses in rat dorsal root ganglion neurons. J. Cereb. Blood Flow Metab..

[111] Brederson J.D., Jarvis M.F. (2008). Homomeric and heteromeric P2X3 receptors in peripheral sensory neurons. Curr. Opin. Investig. Drugs.

[112] Inoue K., Tsuda M., Koizumi S. (2005). ATP receptors in pain sensation: Involvement of spinal microglia and P2X4 receptors. Purinergic Signal..

[113] Hasegawa S., Kohro Y., Tsuda M., Inoue K. (2009). Activation of cytosolic phospholipase A2 in dorsal root ganglion neurons by Ca^2+^/calmodulin-dependent protein kinase II after peripheral nerve injury. Mol. Pain.

[114] Hasegawa S., Kohro Y., Shiratori M., Ishii S., Shimizu T., Tsuda M., Inoue K. (2010). Role of PAF Receptor in Proinflammatory Cytokine Expression in the Dorsal Root Ganglion and Tactile Allodynia in a Rodent Model of Neuropathic Pain. PLoS ONE.

[115] Schäfers M., Svensson C., Sommer C., Sorkin L.S. (2003). Tumor Necrosis Factor-α Induces Mechanical Allodynia after Spinal Nerve Ligation by Activation of p38 MAPK in Primary Sensory Neurons. J. Neurosci..

[116] Bekő K., Koványi B., Gölöncsér F., Horváth G., Dénes Á., Környei Z., Botz B., Helyes Z., Müller C.E., Sperlágh B. (2017). Contribution of platelet P2Y(12) receptors to chronic Complete Freund’s adjuvant-induced inflammatory pain. J. Thromb. Haemost..

[117] Tsuda M., Shigemoto-Mogami Y., Koizumi S., Mizokoshi A., Kohsaka S., Salter M.W., Inoue K. (2003). P2X4 receptors induced in spinal microglia gate tactile allodynia after nerve injury. Nature.

[118] Ulmann L., Hatcher J.P., Hughes J.P., Chaumont S., Green P.J., Conquet F., Buell G.N., Reeve A.J., Chessell I.P., Rassendren F. (2008). Up-regulation of P2X4 receptors in spinal microglia after peripheral nerve injury mediates BDNF release and neuropathic pain. J. Neurosci..

[119] Coull J.A.M., Beggs S., Boudreau D., Boivin D., Tsuda M., Inoue K., Gravel C., Salter M.W., De Koninck Y. (2005). BDNF from microglia causes the shift in neuronal anion gradient underlying neuropathic pain. Nature.

[120] Masuda T., Ozono Y., Mikuriya S., Kohro Y., Tozaki-Saitoh H., Iwatsuki K., Uneyama H., Ichikawa K.I.H.U.R., Salter M.W., Tsuda T.M.Y.K.H.T.-S.M. (2016). Dorsal horn neurons release extracellular ATP in a VNUT-dependent manner that underlies neuropathic pain. Nat. Commun..

[121] Schüpke S., Neumann F.-J., Menichelli M., Mayer K., Bernlochner I., Wöhrle J., Richardt G., Liebetrau C., Witzenbichler B., Antoniucci D. (2019). Ticagrelor or prasugrel in patients with acute coronary syndromes. N. Engl. J. Med..

[122] Dong J., Wang F., Sundararajan S. (2020). Use of Dual Antiplatelet Therapy Following Ischemic Stroke. Stroke.

[123] Stock T.C., Bloom B.J., Wei N., Ishaq S., Park W., Wang X., Gupta P., Mebus C.A. (2012). Efficacy and Safety of CE-224,535, an Antagonist of P2X_7_ Receptor, in Treatment of Patients with Rheumatoid Arthritis Inadequately Controlled by Methotrexate. J. Rheumatol..

[124] Keystone E.C., Wang M.M., Layton M., Hollis S., McInnes I.B. (2012). Clinical evaluation of the efficacy of the P2X_7_ purinergic receptor antagonist AZD9056 on the signs and symptoms of rheumatoid arthritis in patients with active disease despite treatment with methotrexate or sulphasalazine. Ann. Rheum. Dis..

[125] Eser A., Colombel J.F., Rutgeerts P., Vermeire S., Vogelsang H., Braddock M., Persson T., Reinisch W. (2015). Safety and Efficacy of an Oral Inhibitor of the Purinergic Receptor P2X7 in Adult Patients with Moderately to Severely Active Crohn’s Disease: A Randomized Placebo-controlled, Double-blind, Phase IIa Study. Inflamm. Bowel Dis..

[126] Bhattacharya A., Wang Q., Ao H., Shoblock J.R., Lord B., Aluisio L., Fraser I., Nepomuceno D., Neff R.A., Welty N. (2013). Pharmacological characterization of a novel centrally permeable P2X7 receptor antagonist: JNJ-47965567. Br. J. Pharmacol..

[127] Letavic M.A., Lord B., Bischoff F., Hawryluk N.A., Pieters S., Rech J.C., Sales Z., Velter A.I., Ao H., Bonaventure P. (2013). Synthesis and Pharmacological Characterization of Two Novel, Brain Penetrating P2X7 Antagonists. ACS Med. Chem. Lett..

[128] Bhattacharya A., Jones D.N. (2018). Emerging role of the P2X7-NLRP3-IL1β pathway in mood disorders. Psychoneuroendocrinology.

[129] Matsumura Y., Yamashita T., Sasaki A., Nakata E., Kohno K., Masuda T., Tozaki-Saitoh H., Imai T., Kuraishi Y., Tsuda M. (2016). A novel P2X4 receptor-selective antagonist produces anti-allodynic effect in a mouse model of herpetic pain. Sci. Rep..

[130] Richards D., Gever J.R., Ford A.P., Fountain S.J. (2019). Action of MK-7264 (gefapixant) at human P2X3 and P2X2/3 receptors and in vivo efficacy in models of sensitisation. Br. J. Pharmacol..

[131] McGarvey L., Birring S., Morice A., Dicpinigaitis P., Pavord I., Schelfhout J., Nguyen A.M., Li Q., Tzontcheva A., Iskold B. (2020). Late Breaking Abstract-Two Phase 3 Randomized Clinical Trials of Gefapixant, a P2X3 Receptor Antagonist, in Refractory or Unexplained Chronic Cough (COUGH-1 and COUGH-2). Eur. Respir. J..

[132] Abu-Zaid A., Aljaili A., Althaqib A., Adem F., Alhalal D., Almubarak A., Aldughaither S., Alghabban S., Alfaraj G., Masoud A. (2021). Safety and efficacy of gefapixant, a novel drug for the treatment of chronic cough: A systematic review and meta-analysis of randomized controlled trials. Ann. Thorac. Med..

[133] Morice A., Smith J.A., McGarvey L., Birring S.S., Parker S.M., Turner A., Hummel T., Gashaw I., Fels L., Klein S. (2021). Eliapixant (BAY 1817080), a P2X3 receptor antagonist, in refractory chronic cough: A randomised, placebo-controlled, crossover phase 2a study. Eur. Respir. J..

